# Understanding AI risks from its characteristics and NMPA regulation perspectives

**DOI:** 10.1186/s13244-026-02283-8

**Published:** 2026-04-27

**Authors:** Yuehua Liu, Wenjin Yu

**Affiliations:** 1 School of Computer Engineering and Science, Shanghai University, Shanghai, China; 2 Shanghai United Imaging Healthcare Advanced Technology Research Institute Co., Ltd., Shanghai, China; 3 Institute for Medical Imaging Technology (IMIT), Ruijin Hospital, Shanghai Jiaotong University School of Medicine, Shanghai, China

**Keywords:** Artificial intelligence medical devices, Artificial intelligence risks, Regulation responses, Full-lifecycle management

## Abstract

**Abstract:**

AI is reshaping medical research and healthcare delivery, yet the translation of AI innovations into clinically approved medical devices remains limited. This article explores the critical role of regulatory frameworks in bridging this translational gap, with a focus on the full-lifecycle supervision model proposed by China’s National Medical Products Administration (NMPA). We first outline the inherent characteristics and risks of AI that challenge conventional evaluation approaches. By examining a patient-centered AI ecosystem encompassing academia, industry, and regulatory bodies, we highlight the misalignment between preclinical AI research output and the relatively small number of approved AI medical devices (AIMDs). In response, we provide a systematic mapping between AI characteristics and corresponding regulatory control measures, offering a point-to-point interpretation of the NMPA’s approach. We argue that effective evaluation must extend beyond performance metrics to include development processes and non-functional attributes such as safety, usability, and explainability. A structured, actionable checklist is proposed to guide the comprehensive assessment of AIMDs throughout their lifecycle. This framework aims to enhance regulatory clarity, promote safe deployment, and ultimately improve public trust and patient outcomes in the era of AI-powered medicine.

**Critical relevance statement:**

This framework aims to improve regulatory clarity, supporting safe deployment of AI medical devices, enhancing public trust, and ultimately optimizing patient outcomes in AI-powered healthcare.

**Key Points:**

- Despite the rapid AI advancement, the number of approved AI medical devices remains disproportionately small, revealing a translational gap.
- The study identifies several intrinsic characteristics of AI that contribute to regulatory complexity and potential safety risks in clinical practice.
- A point-to-point mapping is established between AI characteristics and regulatory control measures, providing an interpretation of NMPA full-lifecycle supervision model.
- A detailed actionable checklist is proposed, extending beyond algorithmic performance, thereby promoting transparent and reproducible AIMDs development.
- The framework provides a policy-relevant pathway for harmonizing AI innovation with regulatory oversight, fostering patient-centered integration of AI into healthcare.

**Graphical Abstract:**

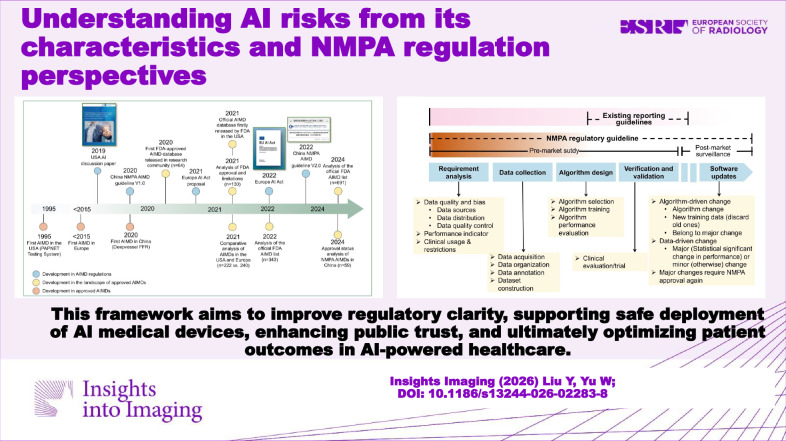

## Introduction

The integration of AI has been changing human life in many ways, especially in the healthcare sector. While the benefits of AI are evident in preclinical settings, its real-world capabilities and limitations still remain to be assessed [1]. Regulation agencies play such a role by assigning a certification to the artificial intelligence medical devices (AIMDs) after a process of stringent review. Medical devices are regulated by country-specific laws and regulations such as the U.S. Food and Drug Administration (FDA), European Union (EU) Medical Device Regulation (MDR) and China National Medical Products Administration (NMPA). As a result, the FDA has already approved 950 AI/ML-enabled medical devices [2]. Accordingly, by 2020, over 240 AIMDs had been authorized in Europe, and as of 2023, 59 AIMDs had been authorized in China, as reported in our previous studies [3, 4]. Such increases in the number of AIMDs imply the pressing demand for the integration of AI into practical clinical settings.

However, it can be observed that the number of AIMDs does not match the efforts in AI research approaches. The main factors hindering translation of AI into practice are the credible concerns that AI algorithms always suffer from uncertain, unexplainable issues and so on. Numerous studies have highlighted these implementation challenges, including the lack of data diversity [5], bias [6], inaccurate metrics [7, 8], generalization gap [9, 10], fairness [11] and so on. The issues are fundamentally rooted in the very characteristics of AI. In fact, each can be traced back to different aspects of how AI is designed, developed and evaluated. There are further concerns that the model might be evaluated on inappropriate testing datasets, creating an “illusion of progress.”

Despite widespread acknowledgement that AI algorithms face bias and safety concerns, there remains a shortage of a systematic and high-level complete review of challenges when using AI and guidance on how to mitigate these risks. To address this gap and fully understand the barriers that may be present between preclinical scenarios and practical clinical settings, this work starts by exploring the risks from AI characteristics perspective. While there are already a few studies on the EU AI Act [12] and the FDA’s AI regulatory framework [13], the interpretation of China’s NMPA regulations remains largely unexplored. We then try to map these risks on the NMPA AIMDs guideline, and provide guidance on how to apply the principles from the standpoint of regulation, ensuring AI operates in an evidence-based, ethical and safe manner. Given that regulation is the final pass for an AI algorithm to be a commercial AI product, it should consolidate the AI risks and offer mitigation measures as possible. Notably, the NMPA’s AIMDs guideline introduces a comprehensive, whole lifecycle supervision model, which closely echoes the regulatory perspective highlighted in a recent publication on the FDA’s AI oversight [13]. This convergence suggests a growing international alignment toward full-lifecycle governance in the regulation of medical AI.

## AI introduction

### Definition and types of AI

**Fig. 1:**
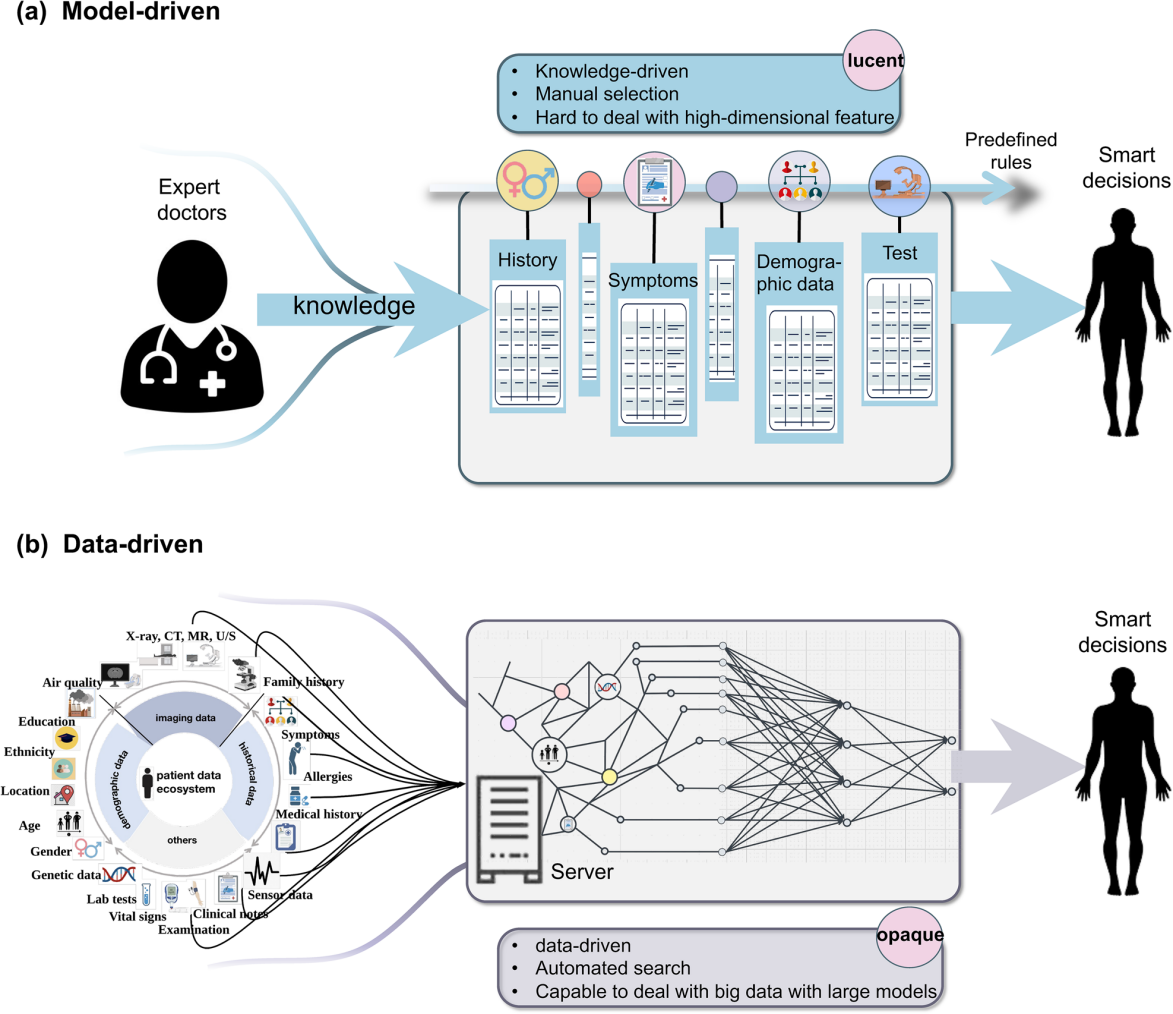
Working principles of (**a**) model-driven and (**b**) data-driven AI algorithms. The rapid development of large models, initially famous with large language models (LLMs) such as ChatGPT, represents a pivotal milestone in the history of AI. Recently developed multi-modality LLMs excel at discovering intricate structures in a wide variety of data formats, such as images, voice, text, genes, molecules, time series, and so on

AI was coined at Dartmouth and proposed by Professor John McCarthy in ‘The Dartmouth Summer Research Project on Artificial Intelligence’ [14]. With the impressive progress since then, AI algorithms can be mainly classified into data-based and model-based types (as shown in Fig. 1).

### Characteristics of AI

#### Explainability

Although the rapid progress of AI facilitates huge breakthroughs in performance in various applications, the models are becoming more powerful but less interpretable and explainable. If clinicians cannot understand the decision-making, they might be violating patients’ rights to informed consent and autonomy [15, 16].

#### Reliability

AI systems face unique attacks, and these attacks are aimed at either data or systems. The attacker can learn and identify the weakness of the AI system, and make a very small change to the input, which might make no difference for humans but is enough for AI systems to make incorrect decisions.

#### Privacy

The development of AI medical devices requires the availability of a massive amount and diverse types of data, which may need to be collected from or even possibly shared across multiple institutions. The sensitive data must be anonymized and deidentified. Due to its high risk in the consequences of disclosures, the notions of cybersecurity and privacy may need to be reimagined entirely [17].

#### Bias

**Fig. 2:**
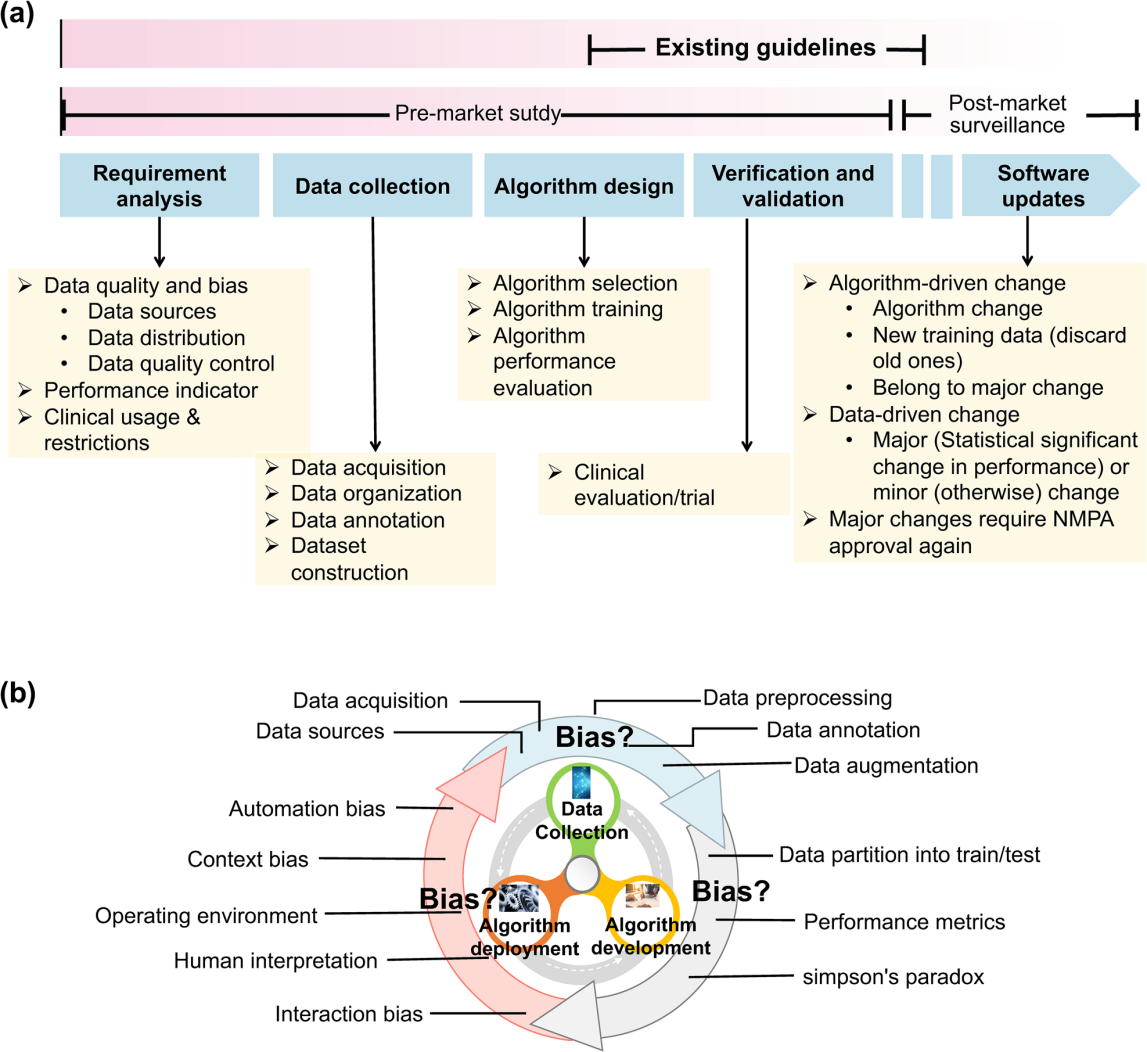
(**a**) NMPA full-lifecycle supervision model on AIMDs and (**b**) the embedding bias sources

AI solutions are not just a technique themselves, but rather involve a lifecycle including data, algorithms and the user interaction loop [18]. AI solutions are vulnerable to biases that might happen at any possible stage, resulting in “unfair” decisions. As shown in Fig. 2, during the data collection phase, inadequate training data or samples that are not representative of the target population can result in selection bias, leading to systematic performance degradation when the model is applied to broader or different clinical populations. In addition, measurement or labeling bias may be introduced when data acquisition protocols, preprocessing pipelines, or annotation standards are inconsistent across institutions or over time. Such heterogeneity is common in multi-center medical datasets and can propagate uncertainty and error into downstream model predictions. Furthermore, class imbalance, where one class or subgroup is substantially underrepresented, is a frequent issue in medical AI due to low disease prevalence, and may result in elevated false-negative rates for clinically critical conditions.

**Fig. 3:**
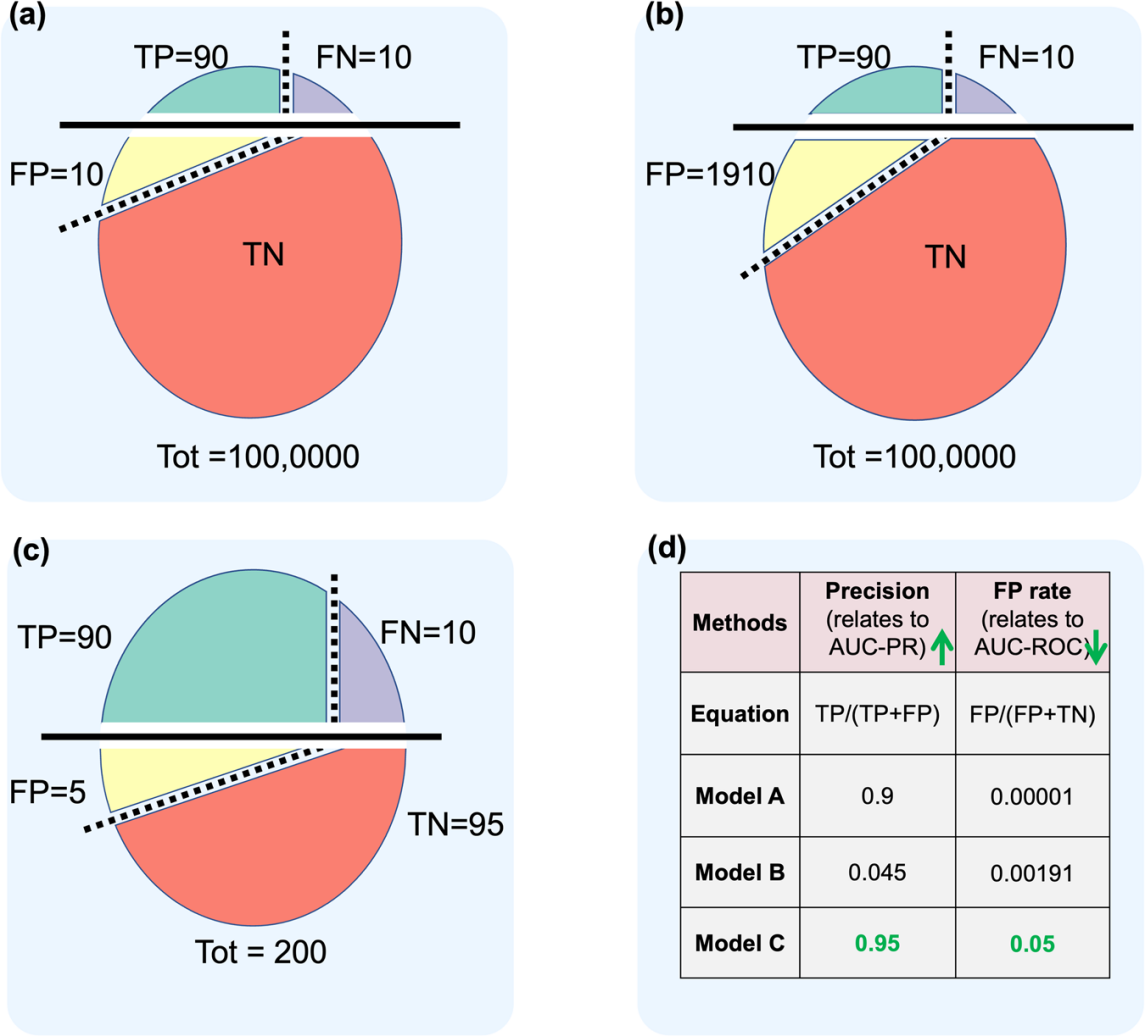
Three diverse cases of testing datasets. **a**–**c** show the prediction results from model A to C, and **d** lists the precision and FP rate of each model. Green arrow “↑” and “↓” indicates the higher the better and the lower the better. Given the model accuracy “model A > model B > model C,” precision does not always reflect the true capability of the model, since according to the precision metric, Model C is better than both Model A and Model B instead. While the FP rate is always in line with the real performance and Model A is assigned with the smallest value

At the stages of algorithm development and deployment, bias may be further amplified by inappropriate model evaluation strategies, particularly when global performance metrics are used without consideration of class imbalance or in low-prevalence settings. Figure 3 shows a metric bias example from AUC-ROC vs. AUC-PR. Beyond evaluation, one of the most common and consequential sources of bias arises from distribution shifts. These shifts may be caused by changes in patient populations, disease prevalence, clinical workflows, or diagnostic technologies, and can progressively degrade model performance after deployment if not actively monitored. Importantly, bias in deployed AI systems is also influenced by the human–AI interaction loop. Changes in clinician behavior, or inconsistent adoption across users and settings, may introduce additional sources of systematic error that are not captured during model development.

#### Fairness

Fairness is defined as a recently developed statistical field that formalizes minimizing disparate treatment and impact via quantifiable fairness criteria, which is to evaluate differences in performance metrics (e.g., accuracy, true positive rate, false positive rate) across subgroups [19]. Fairness in AI systems is a multi-faceted issue, and various metrics have been proposed to measure different dimensions of fairness, such as demographic parity, equalized odds, predictive parity, equal opportunity and so on [20, 21]. However, these metrics are often mutually incompatible, creating trade-offs that must be explicitly considered in regulatory evaluation. According to the well-established impossibility theorem [22], no single model can satisfy all fairness criteria when base rates differ across groups. For example, while demographic parity ensures equal selection rates across groups, equal opportunity focuses on equal true positive rates.

#### Adaptive/dynamic

With the explosive amount of data, the current adopted AI techniques for medical applications are dominated by data-driven approaches. Since data can be generated in a real-time manner, there has developed a specific technique (i.e., online learning) to handle this scenario. This leads to another unique characteristic of AI algorithm, which is called adaptive or dynamic.

#### Reproducibility and repetitiveness

From the model development level, i.e., model training, reproducibility or repetitiveness is the ability to reproduce identical models by backing up all the information that is possibly needed in every model development stage, so that the model can be reproduced later by different stakeholders. In this sense, even the same dataset, the same code base and the same algorithm might generate a different model, particularly for deep learning algorithms, due to the stochastic/uncertain/random nature of them. From the results generation level, i.e., model testing, reproducibility or repetitiveness refers to the ability of a trained model to generate the same result when we try multiple times.

## AI in medical research enterprise

**Fig. 4:**
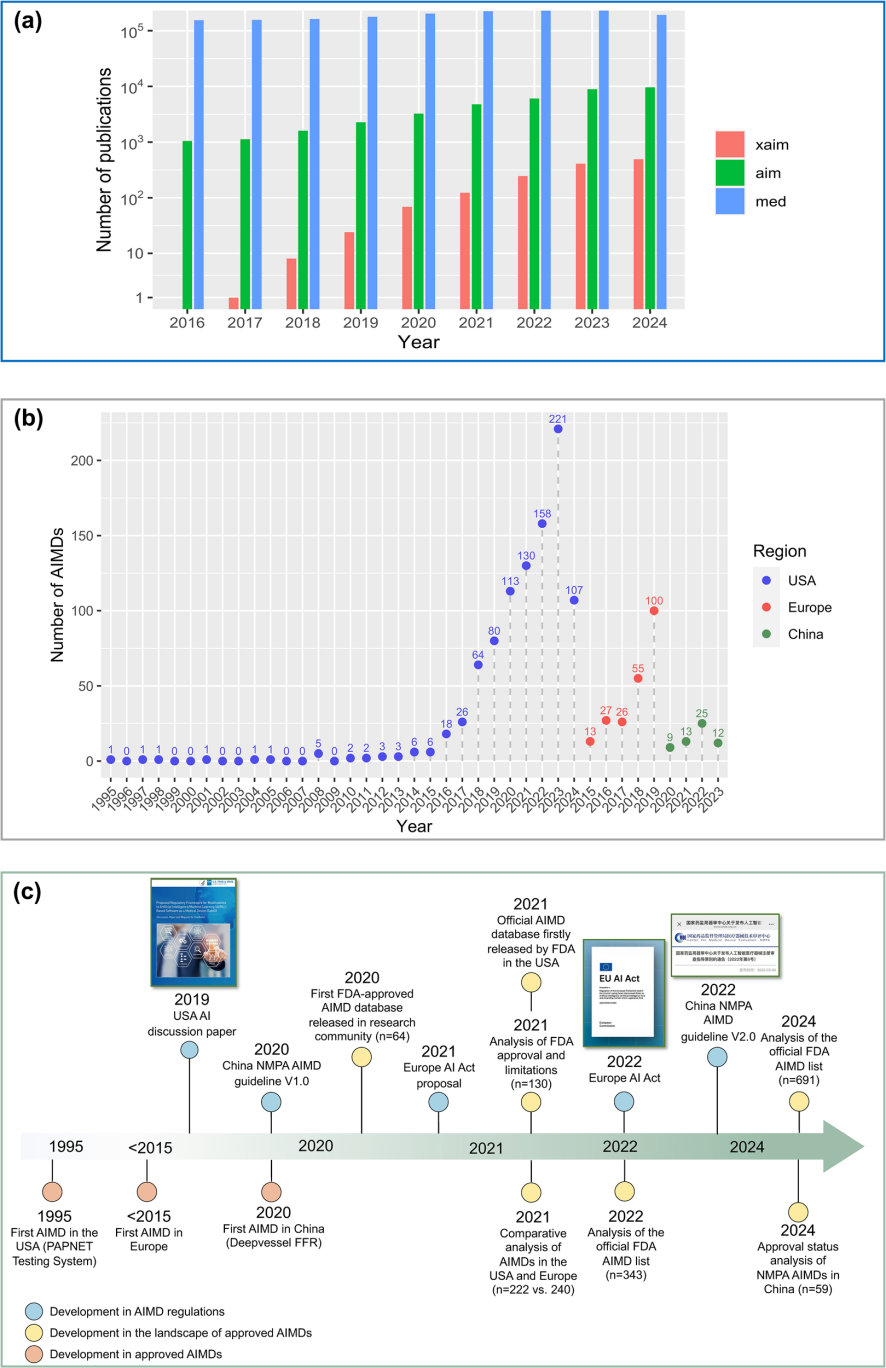
Overview in academia, industry and regulatory sectors regarding AI in medical research enterprise. **a** Timeline showing the number of publications on medical artificial intelligence per year, found by using the same search criteria on Scopus. Specifically, the exact Scopus search strings for “xaim,” “aim” and “med” are TITLE-ABS-KEY(“explainability artificial intelligence medical”), TITLE-ABS-KEY(“artificial intelligence medical”) and TITLE-ABS-KEY(“medical”), respectively. The search was undertaken on October 30, 2024. **b** Number of approved (USA, China) and CE-marked (Europe) AIMDs. The data for the USA and China are extracted from the official website [2] and our previous study [4]. Noted that since only the number between 2015 and 2019 is available for Europe, we will leave the number outside this time scope as NA (not applicable). Unlike FDA-approved AIMDs, there is currently no dedicated or centralized public registry for AIMDs with CE marking in Europe. As a result, EU-related statistics largely rely on secondary sources, such as academic surveys and literature-based reviews. To the best of our knowledge, the most recent comprehensive academic studies systematically summarizing CE-marked AI medical devices only cover data up to 2020. **c** Timeline of the progress of AIMDs regulations (blue circles), compared with approved AIMDs (orange circles) and its landscape (yellow circles) in the USA, Europe and China

The progress and recent success of AI in medical research have largely contributed to the collaboration in the entire research enterprise. The main constituents of AI medical research enterprise are patients, academic research centers, industry sectors, governmental regulatory authority bodies and payers. All of them are working together toward a more patient-centric enterprise. However, the developed approaches largely failed to be adopted in real medical practice [23]. As seen in Fig. 4, across the global market, there are already several analyses on the landscape of government-approved AIMDs specific to different countries, such as Europe, the US, China, and so on [3, 4, 24–26].

Legal regulation plays a significant role in regulating medical devices, and regulations particularly for AI are reinforced with the prosperity of AI technologies across the globe. The FDA released a discussion paper in 2019 trying to regulate medical devices embedding AI applications, which heralds a new era of AI regulation across medical devices [27]. On April 21, 2021, the EU Commission published its proposal for an AI Act [28]. The EU AI Act (in fact specified in an EU regulation 2024/1689) has just officially been approved on 13 March 2024 [29], which is claimed as the first comprehensive legal framework for AI in the study [12]. NMPA published the first Chinese guidelines for AIMDs in 2021, followed by an updated, improved version, “AIMDs review guideline” to replace the original one, “key review point for deep learning assisted decision-making medical software” in 2022 [30]. A more comprehensive and comparative illustration can be found in our previous study [4], including the aspects in regulatory pathway, government-released AI-specific documents, reference link to approval AIMDs, AIMDs regulatory definitions, AIMDs regulatory classifications and so on.

## What is NMPA AIMDs guideline?

In the context of NMPA AIMDs review guideline, this guideline aims to guide registration applicants in establishing the lifecycle process and preparing the registration submission materials for AIMDs. It also standardizes the technical review requirements and provides references for the system inspection of AIMDs. The full lifecycle includes not only the evaluation stage that most existing guidelines focus on, but also the early phase of product development, where requirements on dataset and algorithm information should be fulfilled as well. To be more concrete, NMPA AIMDs regulatory guideline asks for the supervision from requirement analysis, data collection, algorithm design, validation and verification, and software update (see Fig. 2). To operationalize the NMPA AIMDs review guidelines, we constructed a checklist summarizing the main regulatory considerations (Table 1).

**Table 1:** NMPA AIMDs review guideline checklist

Item number	Theme	Recommendation
1	Algorithm general information	Describe the name, type, structure, input/output, flowchart, framework, operating environment of the algorithm and specify the rationale.
2	Algorithm risk management	Identify the risk level of software (minor/moderate/major).
		Specify the mitigation measures regarding possible risks (e.g., underfitting/overfitting, false negatives/positives, data contamination and bias).
		Consider risks for imported software, e.g., race, epidemiological features, and clinical diagnosis and clinical treatment difference.
3	Algorithm requirement specification	Key considerations on data acquisition (e.g., data source compliance requirement, data source sufficiency and diversity requirement, data distribution scientific and rationality requirement, data quality control requirement), algorithm performance and usage restrictions.
4	Data quality control	Describe the data acquisition process, including acquisition equipment and process (including personnel responsibility, acquisition steps, result examination), de-identification and so on.
		Describe the data organization process, specifying data cleaning and preprocessing methods.
		Describe the data annotation process, including annotation resource management, annotation process, and annotation quality evaluation.
		Describe the data augmentation process (if applicable), specifying augmentation objects, methods, ratio and so on.
		Provide data distribution in terms of target disease epidemiology information after above each step.
		Provide the information of public dataset (e.g., name, creator, data size) and usage information.
5	Algorithm training	Provide data distribution of training dataset, tuning dataset (if applicable)
		Specify the evaluation indicators, training methods, training objectives, and tuning methods (if applicable)
		Provide ROC curve, confusion matrix, Curves of training amount of data over evaluation indicators (to justify data sufficiency), etc.
6	Algorithm verification and validation	Provide data distribution of the testing dataset
		Provide false-negative rate and false-positive rate, repeatability and reproducibility, robustness, timeliness or other applicable relevant performance indicator evaluation results
		If a black-box algorithm is applied, provide an analysis report of performance influence factors (e.g., acquisition equipment, acquisition parameters, disease composition, lesion characteristics) to improve the algorithm explainablity (e.g., using subgroup analysis).
		Stress test and adversarial test report (if applicable)
7	Algorithm traceability	Provide an algorithm traceability analysis report, including a traceability table showing the relationships between algorithm requirements, algorithm design, source code, algorithm testing, and algorithm risk management.

For NMPA-approved AIMDs, only a limited number have publicly available submission information, each accompanied by a review summary report. To demonstrate the practical applicability of the proposed checklist, we applied it to an NMPA-approved AIMD “breast ultrasound CAD detection in Yizhun” with publicly available review summary information, and the results are summarized in Table 2. In addition, it should be noted that the checklist assessment is conducted solely based on publicly available review summary information released by the NMPA, rather than the manufacturers’ original submission materials. There is also a scoping review showing the reporting gaps in FDA-approved AI medical devices [31]. Consequently, the analysis in Table 2 is conducted at a high level. Despite this limitation, the results still indicate that the selected AIMD largely fulfills the proposed checklist requirements.

**Table 2:** Application of the proposed checklist to a real-world AIMD

Item number	Checklist	Whether provided in the report
1	Algorithm general information	Yes
2	Algorithm risk management	Yes, risk level is identified as major; risks from false negatives/positives are reported
3	Algorithm requirement specification	Yes
4	Data quality control	Yes, includes data annotation quality control, data diversity analysis, etc.
5	Algorithm training	Yes, provides the evaluation indicators, curves of training amount of data over evaluation indicators, etc.
6	Algorithm verification and validation	Yes, provides sensitivity and specificity, repeatability and reproducibility, timeliness, etc.; provides an analysis report of performance influence factors (including age, acquisition equipment, acquisition parameters, image resolution, disease composition, lesion characteristics, hospital level, etc.)
7	Algorithm traceability	Not disclosed

## AI characteristics implications: insights into China legislation and regulations

### Response to explainability

Explainability can be used interchangeably with interpretability, and they also relate to model transparency. To take a step closer to understand the medical device capabilities, impact factor analysis, in other words, stratification analysis, was proposed in the review guideline as a key review point. This is the most straightforward solution to improve algorithm explainability. Instead of reporting the average performance over the whole testing dataset, it is required to investigate how the algorithm’s performance varies in data subgroups organized by data acquisition device types or protocols, disease subtypes, age, gender, etc. It is essential to study which factor might influence the performance and how big the influence might be. The analysis results could be clarified with use restrictions and necessary warning messages being specified in the instructions for the user. Such an example is an algorithm for Shukun coronary artery stenosis detection. Since there are no sufficient information provided from the NMPA website, we attempted to look for the details in the associated published paper [32], where 45 hospitals were involved for algorithm development and validation. Comprehensive evaluation was undertaken in the sex-based, age-based, geographic-based, detector-row-based and CT-scanner-brand-based subgroups, respectively. Similar examples include deep vessel fractional flow reserve (FFR) in Keya Medical, lung nodule computer-assisted (CAD) detection in Deepwise, intracranial hemorrhage CAD triage in United Imaging, breast ultrasound CAD detection in Yizhun, etc., all of which have conducted impact factor analysis as claimed in their submitted review summary report.

### Response to reliability

When applying AI tools in clinical practice, it is crucial to know the algorithm boundary conditions, i.e., how it performs in rare cases. This concern introduces potential requirements on stress tests and adversarial tests by NMPA. Stress test refers to algorithm performance testing using rare or special real data samples, focusing on evaluating the limitations of the algorithm’s generalization ability. Adversarial test refers to the use of data perturbation, Generative Adversarial Networks or other technologies to generate adversarial samples, and carry out algorithm performance testing using adversarial samples, focusing on evaluating the robustness of the algorithm. Those requirements are not as stringent as other requirements and are listed in the “if applicable” box.

### Response to bias

To prevent data selection bias during the collection stage, multiple data sources need to be ensured regarding data diversity as required by NMPA. The whole data quality control process, which consists of acquisition, preprocessing, annotation and augmentation, has to be standardized to mitigate the possible operation bias caused by the diversity in data collection personnel. Specifically, CMDE has released a standard for regulating data annotation. In addition, it is suggested by NMPA in the review guideline that manufacturers need to guarantee the balance of sample distribution in the training set, and the sample distribution in the tuning and testing sets must conform to the real situation. When AI algorithm is tested, its performance could be controlled by adjusting the testing data. To avoid the assessment bias, NMPA has proposed to establish an “assessment dataset,” which is a concept defined by NMPA and has to be certified before usage. Based on the assessment dataset, all medical devices targeting the same disease could be compared to each other more fairly. The test based on the assessment dataset should be done by an independent test lab qualified by NMPA and the manufacturer has no access to the assessment dataset. Noted that the assessment dataset is a special case of a third-party dataset that fulfills certain requirements. While the use of such assessment datasets is not mandatory under current NMPA guidelines, it is considered a strong plus during AI algorithm evaluation and may enhance the credibility of the AIMDs submission. Since only selected review summary reports of AIMDs are published on the official website, most of them do not explicitly state whether and which assessment datasets were used in the evaluation process. However, the Chinese regulatory body has specifically established a dedicated working group for assessment datasets with the aim of developing versatile datasets to support the review and evaluation of AIMDs.

### Response to privacy and security

A common solution is to utilize privacy computing technologies such as federated learning. It has been highlighted that if any such algorithms are employed, NMPA requires to provide algorithm details. NMPA considers both cybersecurity and data security for AIMDs, which have to showcase their capabilities to deal with network threats such as cyberattacks, data theft, data contamination and so on. More details are elaborated in the NMPA proposed “AIMDs cybersecurity guideline.” Besides the consideration on cybersecurity, data security has been emphasized as an additional key point and needs to be guaranteed during the full lifecycle of AIMDs, covering research, development and post-market phases. All internal activities (including but not limited to data collection, algorithm training, performance evaluation, and software verification) need to be done in a closed or controlled network environment. For all external activities that possibly need to be done in an open network environment (such as data annotation, software validation and so on), manufacturers need to specify the prevention measures from data contamination. If data transfer is required, transfer methods, data contamination prevention measures and data destruction methods need to be provided. In addition, data backups need to be performed for each dataset generated throughout the entire AIMDs production process.

### Response to fairness

Fairness relates to bias and explainability. Assessments of AI models stratified across subpopulations have revealed inequalities in how patients are diagnosed and treated [19, 33]. Algorithmic biases can result in an insufficiently fair AI system and healthcare disparities. Model explainability could be one direction to mitigate biases and improve fairness. In this regard, algorithm fairness also contributes to the improvement of explainability and bias mitigation. Currently, NMPA regulatory documents provide limited guidance on fairness measurement, but the fairness could be qualitatively evaluated through the performance influence factor analysis with sensitive attributes as subgroups. Similarly, although frameworks from the FDA and EU do not have specific regulations dedicated solely to fairness, their fairness requirements are integrated throughout multiple sections, with an increasing emphasis on transparent subgroup analysis and bias audits to ensure consistency [34, 35]. This regulatory gap highlights the need for future Chinese guidelines to better align with international practices in fairness evaluation.

### Response to adaptiveness

Online or adaptive learning would lead to continued changes in the parameters of the AI model. At the present moment, adaptive learning is not allowed when the medical devices are being used in practice, and the algorithm needs to be locked to produce the same results with identical inputs. NMPA requires model modifications to go through the regulatory process for software update registration. The software update is normally divided by the NMPA into minor change and major change according to whether the change would impact the safety and effectiveness of the medical devices. As proposed in the NMPA review guideline, AI software change can be classified into algorithm-driven and data-driven. Algorithm-driven change indicates that the algorithm used has been changed. Normally, all algorithm-driven changes are treated as major changes, and submission to NMPA for regulatory review is required. The data-driven change is due to the inclusion of new training data. The data-driven change type is determined by the change in performance. If the change leads to a statistically significant difference in performance compared with the last registration, it would be considered a major change and vice versa. For minor changes, there is no need for a new submission to NMPA, and it can be managed by the quality management system (QMS) of the manufacturers themselves. In this way, online learning is highly likely to result in a major change that requires a new submission for registration review by NMPA. By contrast, the FDA allows controlled model evolution through predefined change-control plans that keep updates within regulatory oversight.

### Response to reproducibility and repetitiveness

NMPA requires the whole lifecycle process of AIMDs to be traceable and reproducible, thus asking the manufacturers to provide detailed information along the process. In addition, due to the high-risk nature of medical devices, physicians expect both reliable and stable results made by AI. AI systems face challenges in the form of complex environment, the uncertainty of human-machine interactions, variations in technician standards and physician preferences. These might cause significant variation in system performance and lead to unstable or even contradictory results, which results in a loss of patient confidence and satisfaction. Therefore, it is necessary to evaluate the algorithm’s capabilities in producing stable AI-enabled results, i.e., repetitiveness.

## Methodology

We conducted the mapping according to the following methodology. As a first step, we organized the entire guideline document into a list of itemized requirements, and the organization process was performed by both authors, Y.L. and W.Y. Each key requirement was then identified to correspond to a specific characteristic of AI, or as not corresponding to any particular aspect. The identification was conducted by both authors independently to try to obtain a more consistent result. For any identification uncertainties, the authors discussed them with each other in person until a consensus was reached.

## Discussion

Similar to NMPA, the FDA has also laid out the regulatory framework for AIMDs using a product lifecycle approach [27]. Although the specific components involved in lifecycle management are not exactly the same across frameworks, the overarching principles and key elements are broadly similar. A recent study suggests that regulatory approaches in the USA and EU are comparatively more standards-oriented than China’s more rules-based approach [4], as Chinese review guidelines often specify submission requirements more explicitly in a checklist-like format. Despite this, a study [12] indicates that the technical documentation of AIMDs required by the EU is substantially more comprehensive than the documentation needed for US FDA authorization. Notably, there is no absolute standard for determining which regulatory approach is better. Instead, regulators worldwide are pursuing a pluralistic set of strategies and seeking to unlock the full value of AIMDs while ensuring safety. For example, a rules approach might set out a standardized, easier-to-follow pathway for speeding up the review process and easier comparison, while a standards-oriented approach provides manufacturers with more room for innovation, aligning with the US emphasis on fostering innovation [36].

Sharing similar overarching goals with the US FDA and EU AI Act, NMPA also seeks to ensure AI systems’ safety while encouraging innovation. However, an inherent tension exists between these objectives. Ensuring safety often entails extensive documentation and rigorous review, which might discourage manufacturers from incorporating more advanced AI functionalities. Some developers would intentionally limit certain features that could trigger costly regulatory requirements. As mentioned previously, a rules-based approach in NMPA could streamline the review process through a standardized regulation framework, thereby reducing the workload on reviewers and lowering compliance uncertainty for applicants. This prescriptiveness may also help, at least to some extent, mitigate the “lack of clarity” challenges as reported [12] under the EU AI Act. However, it usually requires more complex paperwork for manufacturers. There is a study showing the significant difficulties in medical device companies implementing the EU MDR [37]. With the increasing workload for AIMDs registration review, NMPA also faces the same limitations and practical implementation challenges.

Given the high compliance requirements imposed by NMPA, all medical device companies are affected to varying degrees. While explicit, itemized requirements can improve procedural clarity, the breadth and depth of the documentation and evidence expected may also create a substantial workload, potentially exacerbating practical implementation challenges. This burden is particularly salient for AI start-ups, which often prefer to allocate limited resources to AI model development and validation. In contrast, larger companies are typically better positioned to support dedicated regulatory affairs teams and internal compliance infrastructure, enabling them to navigate and handle regulatory complexity.

Ideally, the NMPA regulatory framework aims to promote the safe deployment. Direct and quantitative evidence in the public domain demonstrating that NMPA’s lifecycle regulatory approach measurably improves AIMD safety is currently limited. However, there are several lines of indirect evidence supporting its safety-enhancing role. Under the NMPA regulatory framework, approval data show stable growth. Besides, a substantial body of academic research has demonstrated that AI models validated in research or retrospective settings frequently exhibit performance degradation when deployed in real-world clinical environments [38, 39]. At the same time, there exists a marked imbalance between the rapidly expanding volume of academic publications on AI-enabled medical software and the comparatively small number of AIMDs that ultimately obtain regulatory approval for clinical use. This disparity suggests that regulatory review frameworks, including that of NMPA, are able to function as a precautionary gatekeeping mechanism, screening out systems that lack adequate evidence of robustness, generalizability, or safety assurance before market entry.

## Conclusion

While it is evident that AI-enabled medical devices enjoy the benefits that AI brings, they also inherently inherit the associated risks. It is observed that the pace at which academic research is conducted is much faster in comparison to AIMDs approvals. With this question in mind, an in-depth investigation is motivated on the gaps between preclinical research to real clinical implementation. Through the mapping of AI characteristics, we further explore how these traits intersect with NMPA AIMDs regulatory guidelines and how the regulatory bodies struggle with the quality control of AI systems.

## Abbreviations

AI: Artificial intelligence
AIMDs: Artificial intelligence medical devices
EU: European Union
FDA: Food and Drug Administration
NMPA: National Medical Products Administration
